# Catalytic Polymerization of Phthalonitrile Resins by Carborane with Enhanced Thermal Oxidation Resistance: Experimental and Molecular Simulation

**DOI:** 10.3390/polym14010219

**Published:** 2022-01-05

**Authors:** Yuxiang Jia, Xiaojun Bu, Junyu Dong, Quan Zhou, Min Liu, Fang Wang, Maoyuan Wang

**Affiliations:** 1 School of Materials Science and Engineering, Key Laboratory of Special Functional Polymeric Materials and Related Technology of the Ministry of Education, East China University of Science and Technology, Shanghai 200237, China; jyuxiang111@163.com (Y.J.); Junyudong@163.com (J.D.); liumin@ecust.edu.cn (M.L.); maoyuanwang2014@163.com (M.W.); 2 Shanghai Great Composites Technology Co., Ltd., Shanghai 201400, China; buxiaojun@sh-grt.com

**Keywords:** phthalonitrile resin, 1,7-bis(hydroxymethyl)-m-carborane, cure, B-H catalysis, oxidation resistance

## Abstract

Biphenyl phthalonitrile (BPh) resins with good thermal and thermo-oxidative stability demonstrate great application potential in aerospace and national defense industries. However, BPh monomer has a high melting point, poor solubility, slow curing speed and high curing temperature. It is difficult to control the polymerization process to obtain the resins with high performance. Here, a BPh prepolymer (BPh-Q) was prepared by reacting 1,7-bis(hydroxymethyl)-m-carborane (QCB) with BPh monomers. The BPh-Q exhibited much better solubility, faster curing speed and lower curing temperature compared with pure BPh and BPh modified with bisphenol A (BPh-B, a common prepolymer of BPh). Thus, the polymerization process of BPh was greatly accelerated at a low temperature, resulting in a BPh resin with enhanced thermostability and oxidation resistance. The experimental and theoretical models revealed the promotion effect of B-H bond on the curing reaction of phthalonitrile via Markovnikov addition reaction due to the special steric structure of carborane. This study provided an efficient method to obtain low-temperature curing phthalonitrile resins with high thermal and thermo-oxidative resistance, which would be potentially useful for the preparation of high-performance cyanide resin-based composites.

## 1. Introduction

As one of the densely cross-linked networks of organic repeat units, phthalonitrile resin provides tunable desired properties through a flexible synthetic [1]. The growing interests in aerospace, national defense industries, UV shielding, corrosion and proton exchange membranes require the development of lightweight phthalonitrile resin with excellent thermostability, superior mechanical property, good flame retardant property and low dielectric properties [1,2,3,4,5]. However, the preparation of phthalonitrile resin remains a big challenge because of the difficulties in controlling the polymerization of pure phthalonitrile monomers and the gelation of the system at high temperature (such as 300 °C) for several days [6].

Several strategies were employed to speed up the polymerization and gelation process in the preparation of phthalonitrile resin. For example, the introduction of small molecule curing additives, such as organic amines, organic acids, phenolic hydroxyl groups and metal salts [7,8,9,10], could catalyze the polymerization of cyano groups by their active hydrogen atoms or metal ions. As a result, the curing time and temperature of phthalonitrile resin were reduced [8,9]. Nevertheless, the instability and low melting point of the small molecule curing additives may cause the voids within the phthalonitrile thermoset and decrease its heat resistance [11,12]. To solve this problem, self-polymerizing phthalonitrile resin was considered [1,13,14]. Cyanide autocatalysis could be effectively promoted by introducing amino or hydroxyl groups into the resin structure [15,16], while the thermal and thermo-oxidative stabilities of the resin were decreased [17,18]. In addition, blending phthalonitrile resin with high performance resins is also a way to improve its curing behavior, such as bismaleimide, novolac and benzoxazine, propargyl reins [19,20,21,22,23]. Cyanide polymerization can be effectively facilitated by the hydroxyl/amino groups produced during the curing process of the blend resins. Although the temperature resistance of these resins is great, it is difficult to further improve the thermal stability of phthalonitrile resin by blending with them. Thus, it was not easy to obtain both accelerated curing process and improved heat resistance performance.

**Figure 1:**
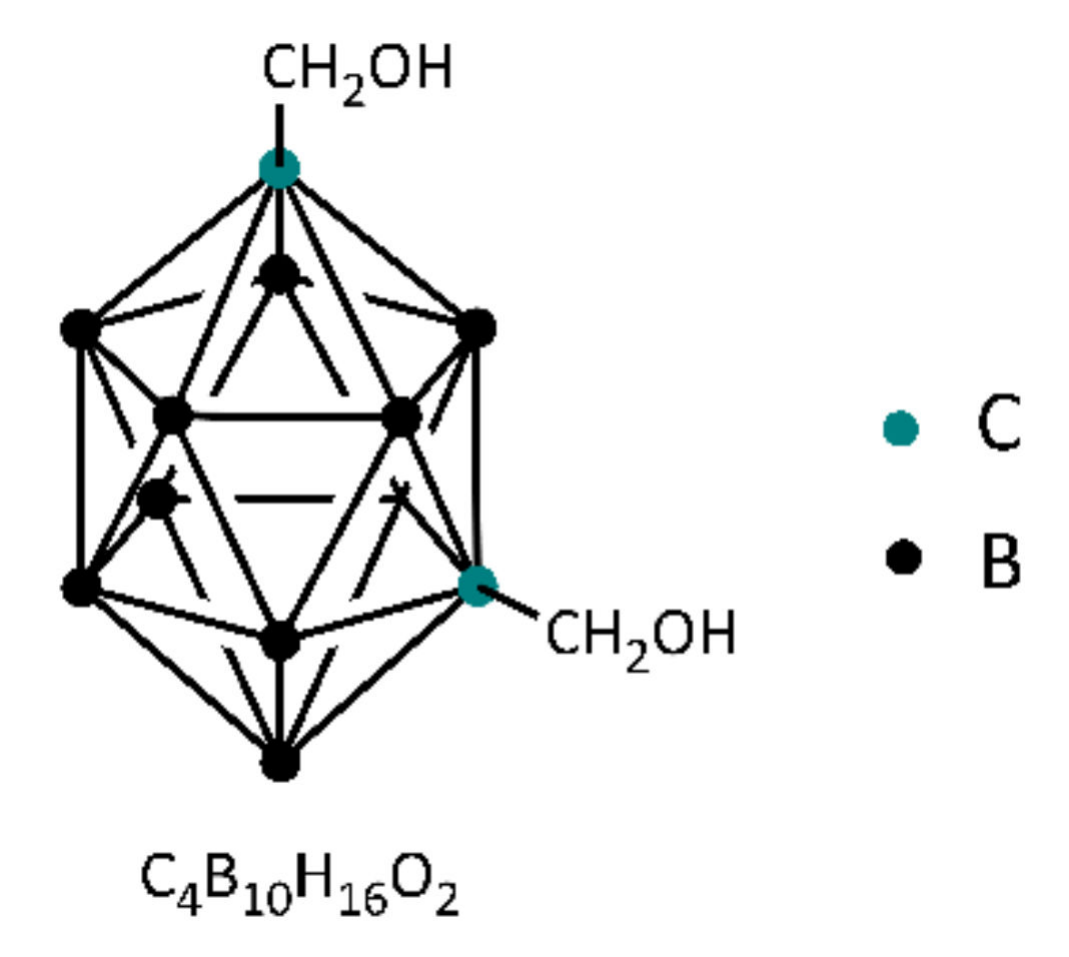
The structure of 1,7-bis(hydroxymethyl)-m-carborane.

Remarkably, the combination of both organic and inorganic constituents opened an efficient way to fabricate phthalonitrile resin [3,24,25]. As a polyhedral cage boron cluster compound, carborane was formed when some boron atoms in the cluster were replaced by carbon atoms [26]. This particular structure endowed the carborane molecules and their derivatives with aromatic properties and good thermal stability [27,28]. 1,7-bis(hydroxymethyl)-m-carborane (QCB) introduces hydroxyl methyl to two C atoms of carborane (Figure 1). The hydroxyl group and B-H of QCB can provide active hydrogen atoms to catalyze the cyanide polymerization reaction, thereby improving the thermostability of phthalonitrile resin.

Here, we designed a BPh prepolymer, BPh-Q, with good solubility, fast curing speed and low curing temperature via the prepolymerization of BPh monomers with QCB. It was found that B-H of QCB could combine with C≡N through hydroboration to accelerate the curing of cyanide group and eventually form dense and uniform thermosets. In addition, the thermosets of BPh-Q also showed high thermal and thermo-oxidative resistance. The present work provided new insights into the improvement of the temperature resistance and the acceleration of the curing process of cyanide resins. This would be useful for the preparation of high-performance cyanide resin-based composites.

## 2. Materials and Methods

### 2.1. Materials

All chemicals and solvents above were used as received without further purification. 1,7-bis(hydroxymethyl)-m-carborane (QCB, Shanghai Titan Technology Co., Ltd., Shanghai, China), bisphenol A (BPA, Sinopharm Chemical Reagent Co., Ltd., Shanghai, China), N-Methyl pyrrolidone (NMP, Shanghai Macklin Reagent Co., Ltd., Shanghai, China) and other solvents (Shanghai Titan Technology Co., Ltd.) were purchased. Biphenyl phthalonitrile (BPh) was synthesized in our lab [29].

### 2.2. Synthesis of BPh-Q Prepolymer

BPh (10.00 g, 22.83 mmol) and QCB (1.00 g, 4.90 mmol) were dissolved in NMP (11.00 g, 110.97 mmol). The reaction solution was stirred at 200 °C for 2 h. The solution was then poured into deionized water slowly, and stirred to precipitate. The precipitate was filtered and washed with deionized water several times, followed by drying at 100 °C for 10 h to afford BPh-Q_10_ as a puce solid powder (10.07 g, yield 91.50 wt%).

BPh-Q_5_, BPh-Q_15_, BPh-Q_20_ and BPh-Q_30_ were prepared via the same route of BPh-Q_10_, in which the addition of QCB was 0.5 g (2.45 mmol), 1.5 g (7.34 mmol), 2.0 g (9.79 mmol) and 3.0 g (14.68 mmol), respectively. The yields of BPh-Q_5_, BPh-Q_15_, BPh-Q_20_ and BPh-Q_30_ were 93.18 wt%, 91.33 wt%, 95.31 wt% and 94.53 wt%, respectively.

### 2.3. Synthesis of BPh-B Prepolymer

BPh (10.00 g, 22.83 mmol) and BPA (1.12 g, 4.90 mmol) were dissolved in NMP (11.00 g, 110.97 mmol). The reaction solution was stirred at 200 °C for 2 h. The solution was then poured into deionized water slowly, and stirred to precipitate. The precipitate was filtered and washed with deionized water several times and dried at 100 °C for 10 h to obtain BPh-B as a green solid powder (10.23 g, yield 92.00 wt%).

### 2.4. Preparation of Thermosets

The curing procedures of BPh-Q (including BPh-Q_5_, BPh-Q_10_, BPh-Q_15_, BPh-Q_20_ and BPh-Q_30_) and BPh-B were determined as:

BPh-Q and BPh-B: 240 °C/2 h, 280 °C/2 h, 300 °C/2 h, 320 °C/2 h, 350 °C/4 h and 375 °C/4 h.

In the air atmosphere, a certain amount of BPh-Q and BPh-B prepolymer powder was cured in a high temperature oven in the procedure described above.

### 2.5. Characterization

Solubility test: the solvent (8 g) and prepolymer (2 g) were added to a lidded test tube. They were then shaken and stood for 2 min to observe whether there was insoluble substance. If there was insoluble matter, the solvent was heated to boiling point and observed to see whether the resin was dissolved. The results were divided into four categories: ++: soluble in room temperature; +: soluble while heating; +−: partially soluble while heating; and −: insoluble.

Thermal gravimetric analysis (TGA) was tested by a NETZSCH 209F1 thermogravimetric analyzer with a heating rate of 10 °C/min (under nitrogen or air atmosphere) and a purge of 40 mL/min, and the experimental temperature range from 50 to 1000 °C. Differential scanning calorimetry (DSC) was performed on NETZSCH 200F3 with a heating rate of 10 °C/min under nitrogen atmosphere and a purge of 50 mL/min from 50 to 400 °C. The thermo-physical properties were determined using a dynamic mechanical thermal analysis (DMA) Q800 analyzer from 50 °C to 500 °C at a heating rate of 5 °C/min and a frequency of 1.0 Hz. The curing procedure was: 240 °C/2 h, 280 °C/2 h, 300 °C/2 h, 320 °C/2 h, and 350 °C/4 h; The size of the sample was polished to 45.0 mm × 10.0 mm × 2.5 mm.

The Fourier transform infrared (FTIR) spectra were recorded with a Nicolet is50 in KBr pellets between 4000 and 400 cm^−1^ in air. The solid-state UV-vis reflectance spectra were analyzed with a UV-vis spectrophotometer (Lambda 950, Varian, America) from 2000 to 200 nm in air. The pyrolyzer/gas chromatograph/mass spectrometer (PY/GC-MS) was performed on an Agilent 7890A-5975C with a cracking temperature of 800 °C. The BPh-Q thermosets were made under the conditions of 350 °C/4 h, with ground powder as the sample. Rheological analyses were performed on a TA Discovery HR-1 from 220 to 400 °C at a heating rate of 2 °C/min. The surface morphology of BPh-Q thermosets was characterized by scanning electron microscope (SEM, Hitachi, Ltd., S-4800, Tokyo, Japan) and energy dispersive spectrometer (EDS, QUANTAX 400-30, Karlsruhe, Germany), and gold spray time was set as 30 s.

### 2.6. Molecular Simulation

The structures of BPh after interacting with QCB were obtained by applying B3LYP method with 6–31 g (d, *p*) basis sets [30,31]. The harmonic vibrational frequencies characterizing the stationary points were evaluated at the same theory level, so that all the obtained structures corresponded to true minima or first order saddle points on the potential energy surface. The electronic energies of all stationary points were calculated by using the M06-2X method, with the def2-TZVP basis sets for each structure previously optimized at the B3LYP/6–31g (d, *p*) level [32,33]. Finally, the relative Gibbs free barriers were determined by combining the calculated electronic energies of the stationary points and the thermal and zero-point energy corrections, as well as the entropy contributions computed at the same level of theory. All calculations were implemented via the Gaussian09 software package [34].

## 3. Results

The main aim of this research was focused on the preparation of a new type of BPh-Q prepolymer with excellent solubility, fast curing speed and low curing temperature. The preparation strategy was based on the reactions of reactive B-H, O-H with C≡N.

### 3.1. Solubility Investigation of BPh-Q and BPh-B Prepolymers

**Figure 2:**
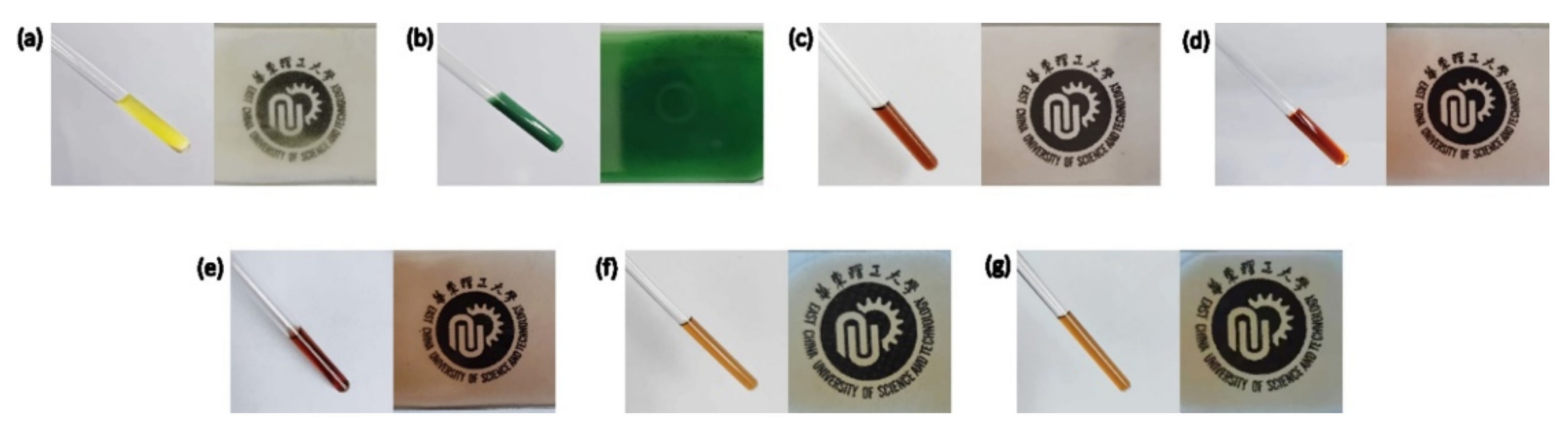
Solubility of (**a**) BPh; (**b**) BPh-B; (**c**) BPh-Q_5_; (**d**) BPh-Q_10_; (**e**) BPh-Q_15_; (**f**) BPh-Q_20_; (**g**) BPh-Q_30_ in THF, the solid content is 20%. Apply the solution on the glass to compare the transparency.

The melting point of BPh monomer is up to 237 °C, which is difficult for the dry prepreg process and melt technologies to meet. At the same time, the poor solubility of resin in low boiling point solvent is difficult for the wet prepreg process and resin solution technologies to meet. Thus, solubility is one of the most important factors to affect the processability in the preparation of resins. Both BPh-B and BPh-Q were readily soluble in solvents with high boiling point and strong polarity such as NMP, DMF and DMAc (Table 1). Furthermore, in solvents with low boiling point such as THF, acetone and CHCl_3_, BPh monomers and BPh-B were partially dissolved even upon the heating treatment. However, all the clear and transparent solutions of BPh-Q indicated its good solubility, which is beneficial for preparing composite prepreg (Figure 2). This solubility improvement may result from the introduction of the QCB cage, in which the strong steric hindrance would destroy the chain regularity of BPh and further decrease the interactions between pendant cyanides on neighboring chains. Thus, more solvent molecules were supposed to diffuse among BPh-Q prepolymers and interacted with them [35].

### 3.2. Curing Behavior of BPh-Q and BPh-B Prepolymers

**Figure 3:**
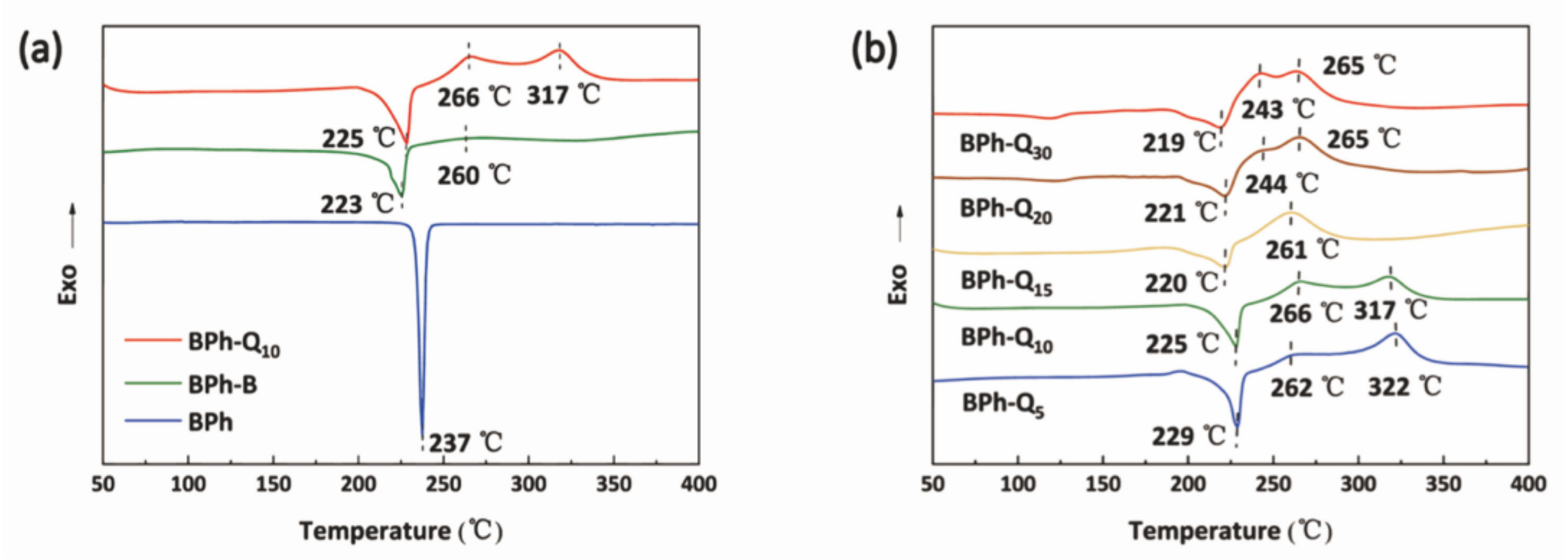
DSC curves of BPh monomers and prepolymers. (**a**) DSC curves of BPh, BPh-B and BPh-Q_10_; (**b**) DSC curves of BPh-Q_5_, BPh-Q_10_, BPh-Q_15,_ BPh-Q_20_ and BPh-Q_30_, the experimental temperature ranges from 50 to 400 °C, with a heating rate of 10 °C/min (under nitrogen atmosphere) and a purge of 50 mL/min.

Curing behavior, generally investigated by DSC, is vitally important for the processing of resins. BPh monomer only exhibited a single endothermic peak around 237 °C due to the melting behavior (Figure 3), suggesting that the self-curing reactions would not occur for individual cyano groups. Since the increasing amount of BPA would decrease the temperature resistance of BPh resin [11], BPA with the same molar ratio as 10 wt% QCB was selected for the prepolymerization with BPh. In case of 10.12 wt% BPA added, the exothermic peak was shifted toward lower temperature (223 °C), followed by the emergence of a weak exothermic peak near 260 °C, indicating that the curing was induced by the reaction between hydroxyl and cyano groups. In contrast, by introducing QCB (5.00 wt%) into the BPh system, there was one endothermic peak at 229 °C and two new exothermic peaks at 262 °C and 322 °C, implying two types of polymerization processes. In addition, BPh-Q has a unique and significantly different melting point from that of BPh monomer and QCB (Figure S1), implying that BPh-Q is a prepolymer rather than a blend of the two substances. With further increased content of QCB, the intensity of exothermic peak at around 262 °C became stronger, while the exothermic peak around 322 °C tended to shift forward gradually. Besides, the enthalpy of curing reaction of BPh-Q_15_ became larger at 260 °C and vanished at 320 °C. In addition, the exothermic peak of 320 °C moved forward to 243 °C in the curve of BPh-Q_20_ and BPh-Q_30_. The results illustrated that the polymerization of cyano groups was greatly accelerated and tended to occur at a lower temperature by increasing the active hydrogen atoms content in BPh-Q prepolymer. In addition, ΔH before and after curing of the prepolymer was also measured to calculate the curing degree (Figure 3b and Figure S2 and Table 2) [36]. It can be seen from Table 2 that the curing degrees of all prepolymers exceed 97%, and the curing is relatively complete.

**Figure 4:**
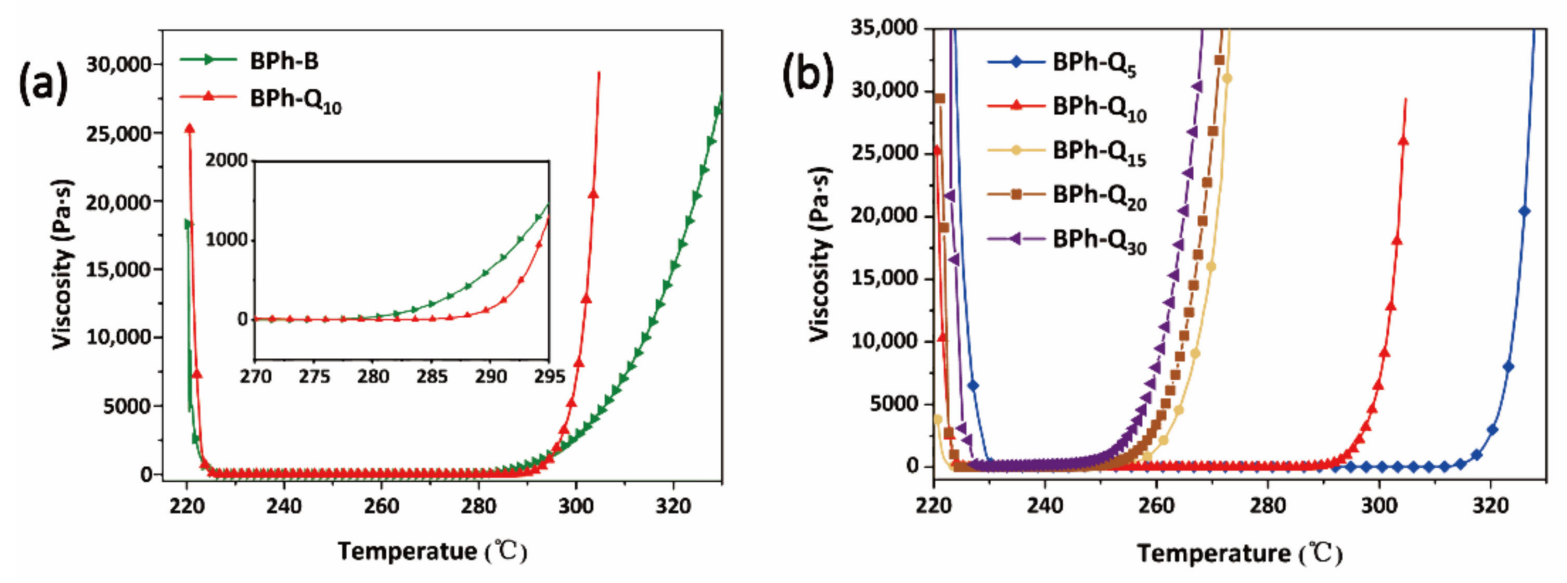
Rheological curves of (**a**) BPh-Q_10_ and BPh-B prepolymers, (**b**) BPh-Q_5_, BPh-Q_10_, BPh-Q_15_, BPh-Q_20_ and BPh-Q_30_, Rheological analyses are performed from 220 to 400 °C at a heating rate of 2 °C/min.

The rheological tests of BPh-Q and BPh-B were subsequently performed to further assess their curing behaviors. Neat BPh monomer showed low viscosity (0.1 Pa·s) up to 400 °C due to the slow rate of its polymerization (Figure S3). With the incorporation of BPA (10.12 wt%), the viscosity of the system decreased dramatically at 220 °C (the minimum viscosity is 0.3 Pa·s), implying the enhanced flexibility of the molecular chain of BPh resin (Figure 4a). Upon heating BPh-B to 277 °C, the viscosity rose gradually, indicating the occurrence of gelation. Compared to BPh-B, the viscosity of BPh-Q exhibited similar behavior at the initial stage (the minimum viscosity is 3 Pa·s), and then increased dramatically at around 287 °C (10 °C higher than BPh-B), affording an extended processing window. In general, the rising slope of the rheological curve after gelation could indirectly reflect the curing rate [37]. Therefore, the sharp slope of BPh-Q_10_ above 287 °C further confirmed the accelerated polymerization by QCB. Furthermore, as the QCB content was increased from 5 to 30 wt% (Figure 4b), the processing window of BPh-Q was significantly narrowed and the temperature corresponding to the appearance of gelation decreased accordingly. The results indicated that the polymerization degree of BPh-Q prepolymer was improved with increasing QCB content under the same experimental conditions.

### 3.3. Curing Mechanism of BPh-Q Prepolymer

**Figure 5:**
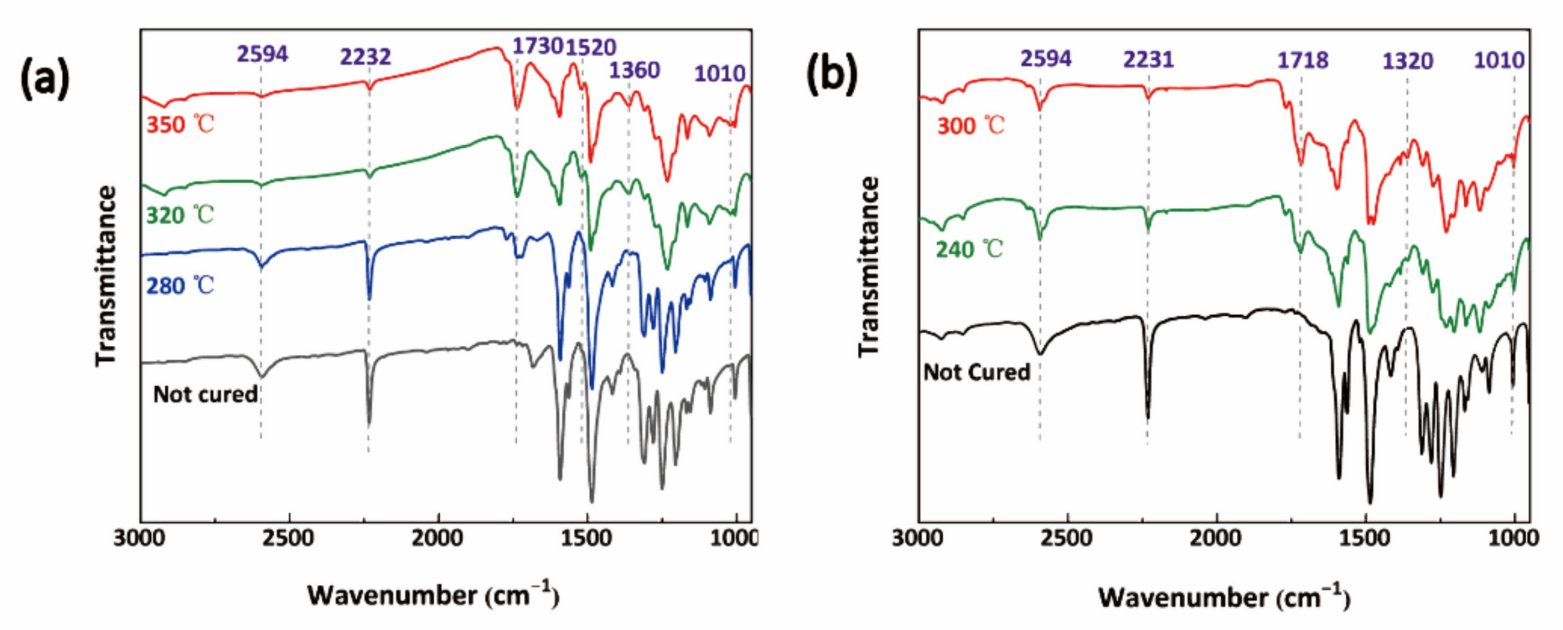
FTIR spectra of (**a**) BPh-Q_10_ prepolymer (**b**) BPh-Q_30_ prepolymer. The FTIR spectra are recorded between 4000 and 400 cm^−1^ in air.

**Figure 6:**
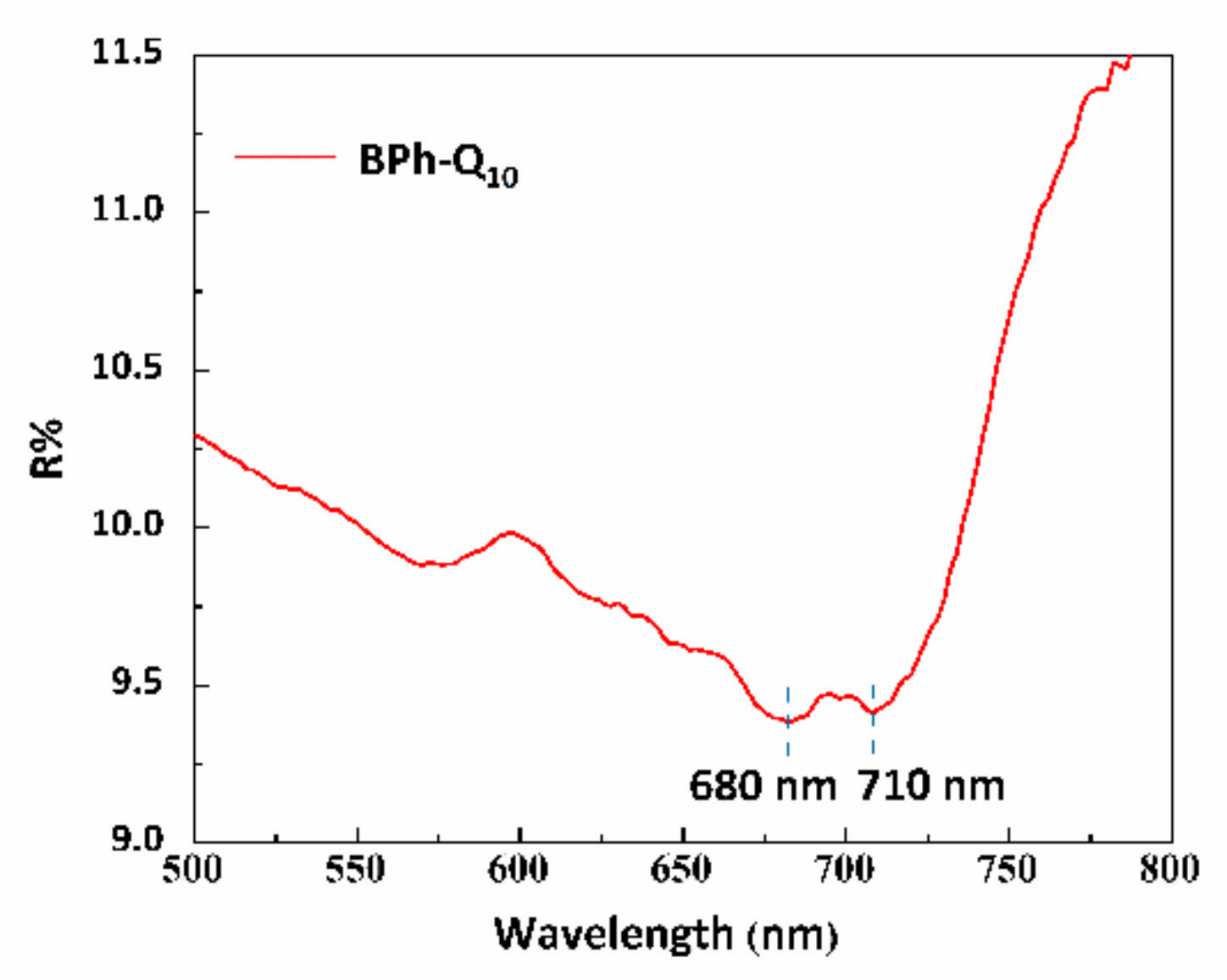
UV-Vis spectrum of BPh-Q thermosets. The UV-Vis reflectance spectrum is analyzed from 2000 to 200 nm.

**Scheme 1:**
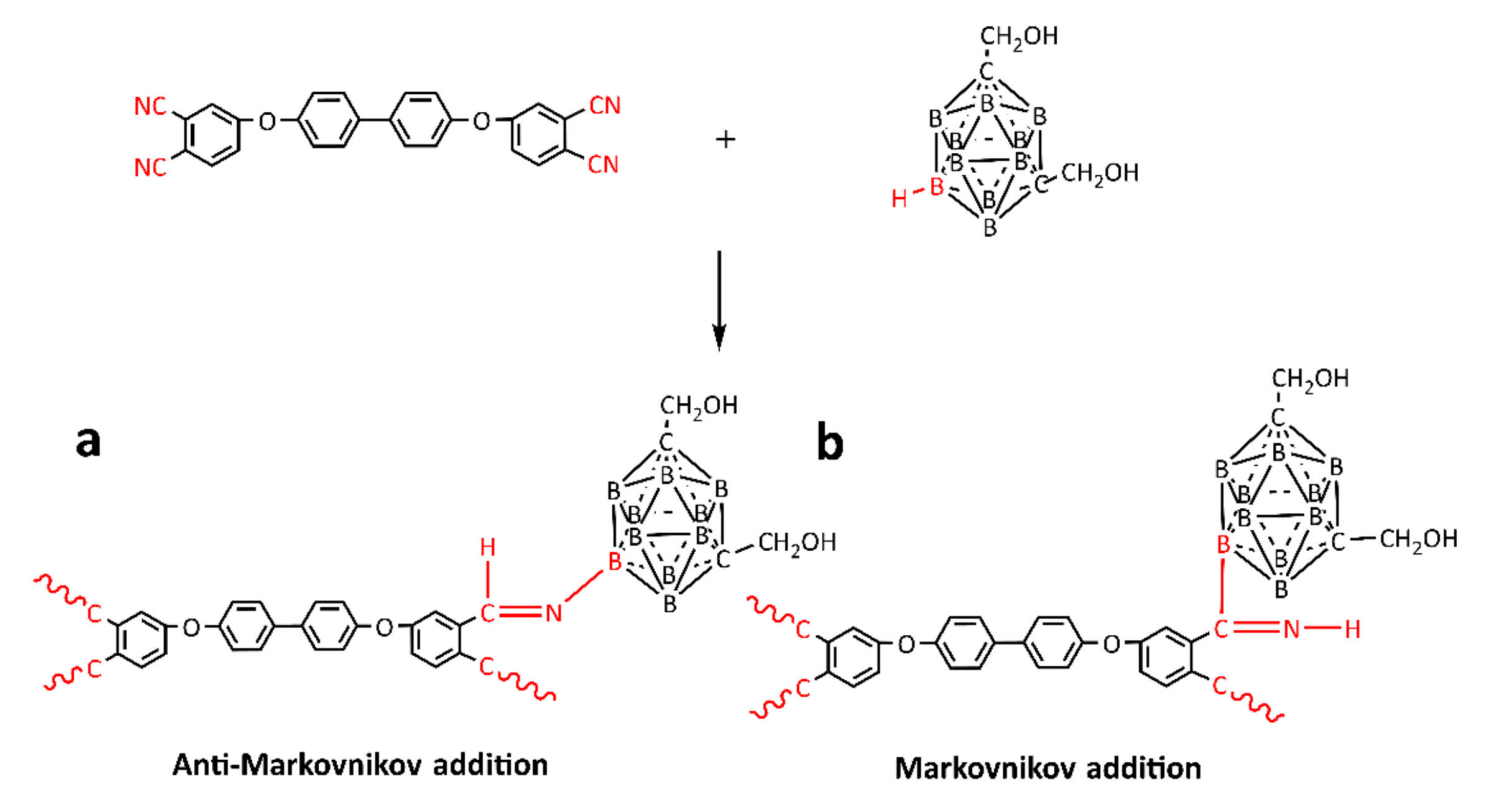
(**a**) Anti-Markovnikov addition and (**b**) Markovnikov addition of BPh-Q.

FTIR was used to analyze the structural transformations of BPh-Q_10_ and BPh-B during the curing processes. For BPh-Q_10_ (Figure 5a), the peak observed at 2232 cm^−1^ was ascribed to the cyano stretching group, and it was evidently weakened as the curing process proceeded. After curing at 280 °C, isoindoline structures were formed as confirmed by the appearance of the peak at 1730 cm^−1^ [1,38,39]. Furthermore, after curing at 320 °C, the peaks at 1360 cm^−1^ and 1520 cm^−1^ related to the stretching of the triazine ring were detected [9]. Meanwhile, a weak absorption band at 1010 cm^−1^ was assigned to the stretching vibration of phthalocyanine ring [40,41]. The phthalocyanine rings formed during the curing reaction of BPh-Q_10_ was further confirmed by UV-vis spectra (Figure 6). Two reflection peaks at 680 nm and 710 nm were ascribed to the Q-band characteristic peaks of the phthalocyanine rings [28]. Moreover, no obvious change was observed for the FTIR spectrum of BPh-Q_10_ cured at 350 °C compared with that at 320 °C. The exothermic peak at around 317 °C in DSC curve of BPh-Q_10_ belonged to the curing reaction of triazine and phthalocyanine rings, while the exothermic peak at 266 °C was related to the formation of isoindoline structures (Figure 3a). In addition, the peak at 2594 cm^−1^ related to the stretching of B-H bonds in QCB was obviously weakened during the curing, indicating the curing reaction of B-H bonds. Furthermore, the absorption peak of methylene at 2920 cm^−1^ indicated that the C-H bonds might be formed by the anti-Markovnikov addition reaction of B-H and cyanide groups (Scheme 1a) [42,43,44,45].

In contrast, the cyano absorption peak of the FTIR spectra of BPh monomer remained unchanged in the curing process. Moreover, the intensity of 2232 cm^−1^ (cyano stretching) in the spectra of BPh-B diminished with increasing temperature, indicating the catalyzed polymerization of BPh monomers by the incorporation of BPA (Figure S4). However, compared with that for BPh-Q_10_, the intensity of phthalocyanine ring (1005 cm^−1^) in the spectra of BPh-B was enhanced while the intensities of triazine ring (1520, 1360 cm^−1^) and isoindoline (1722 cm^−1^) were weakened. Since the phthalocyanine ring showed a green color [46], it could be concluded that the phthalocyanine rings were generated in a large quantity during the polymerization process of BPh-B.

Furthermore, for BPh-Q_30_, after curing at 240 °C, the peak strength of the cyano group (2231 cm^−1^) decreased significantly, and the characteristic peaks of triazine ring and isoindoline ring appeared at 1320 cm^−1^ and 1718 cm^−1^, respectively (Figure 5b). In addition, with the increase of curing temperature, the absorption peak of isoindoline ring became larger than the thermosets cured in 240 °C. Combined with Figure 4b and Figure 5a, it was obvious that the formation temperature of triazine and phthalocyanine rings was gradually shifted from 320 °C to 243 °C with the increasing amount of QCB (from 5 to 30 wt%).

**Figure 7:**
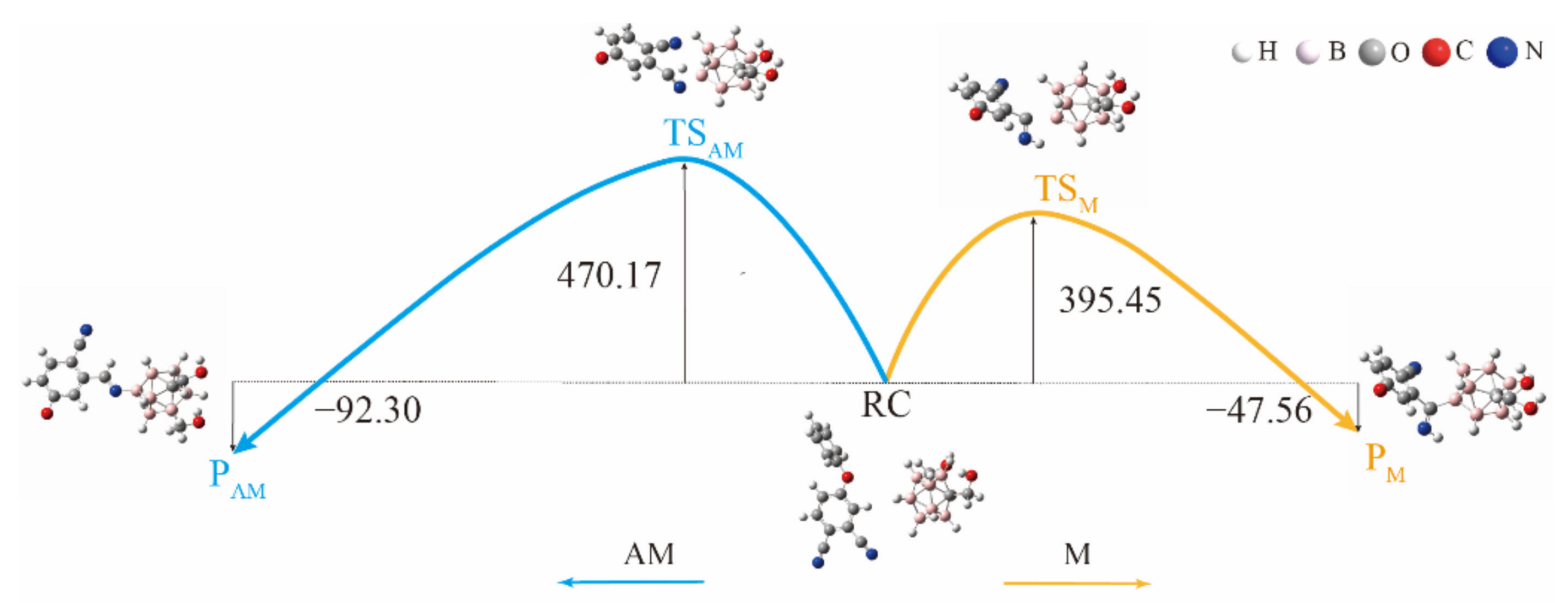
Two possible pathways of hydroboration of BPh with QCB (the blue line indicates path AM; the yellow line is for path M; the energies are given kJ/mol), and products (PAM and PM) of the two pathways are also shown. All calculations are implemented via the Gaussian09 software package.

In order to further explore the hydroboration of B-H with C≡N groups, the molecular calculations were performed. From the calculation results, two possible pathways were found for the addition reaction of BPh and QCB (Figure 7). The barrier height of path AM (for an anti-Markovnikov addition reaction, the B atom was connected with the N atom, Scheme 1a) was 470.17 kJ/mol, which is higher than that of path M (395.45 kJ/mol, for a Markovnikov addition reaction, the B atom was connected with the C atom, Scheme 1b) [47]. Besides, the bond lengths of C-N bond and B-H bond in TSM were 1.17 and 1.45 Å, respectively, which were longer than in TSAM (1.28 and 2.59 Å), confirming that these two bonds were greatly weakened in TSM (Table S1). Thus, path M was the more favorable reaction channel. In other words, the addition reaction of BPh and QCB mainly occurred in the way of Markovnikov addition reaction. In combination with the FTIR results, it could be inferred that, during the curing process, BPh-Q_10_ firstly produced a large number of N-H bonds and then formed ionized intermediates to promote the cyanide polymerization reaction. With gradually increasing temperature of the polymerization, the energy required for AM was sufficient, leading to the formation of C-H bonds through anti-Markovnikov addition reaction of the remaining cyanide groups and QCB.

**Scheme 2:**
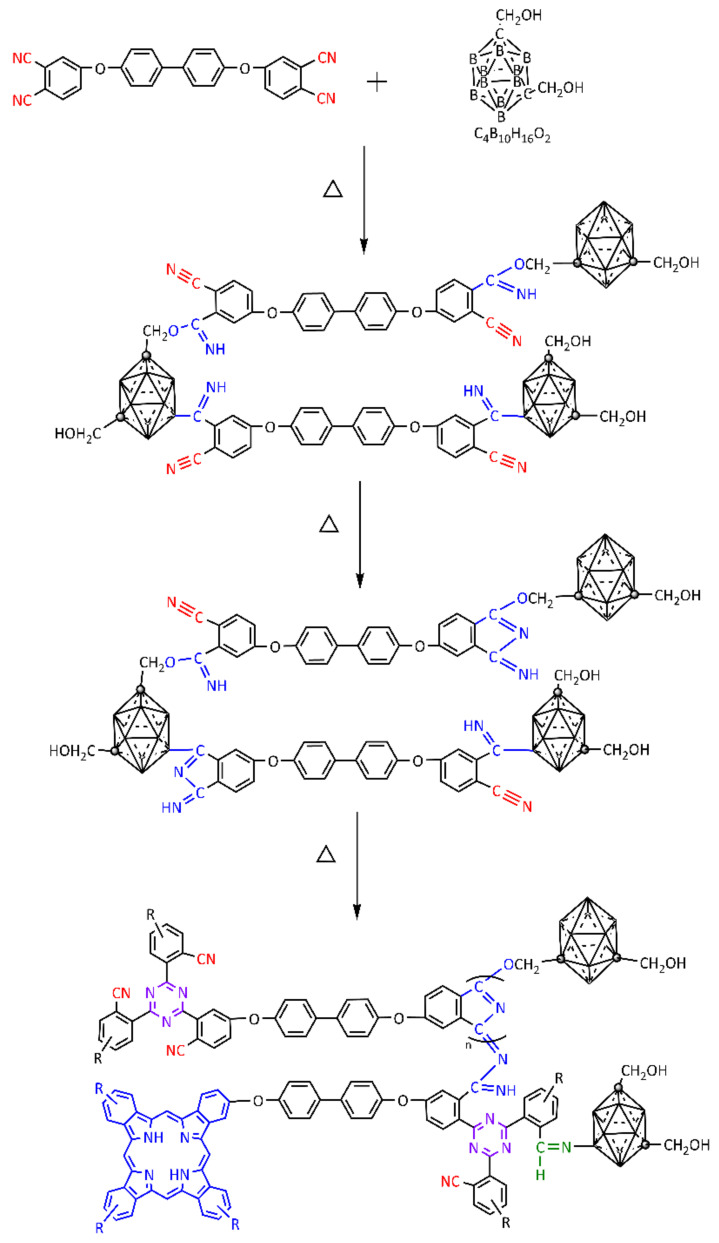
The possible polymerization route and structures of BPh-Q: the initiation of nitrile groups, the ringforming polymerization of nitrile groups and the possible structure of the resultant polymers.

Based on all these results, the mechanism of the curing reaction of cyanide catalyzed by hydroxyl and B-H groups in QCB was illustrated in Scheme 2. The curing reaction of BPh was intrinsically the nucleophilic reaction of the cyano groups. The hydroxyl and B-H groups in QCB provided reactive hydrogen atoms to attack the cyano groups, which separated the positive and negative electron centers of the cyanide groups and further formed the electronegativity centers. Moreover, the hydroxyl groups and the active hydrogen atoms on B-H were transferred to N-, thereby triggering a continuous nucleophilic reaction and forming triazine ring structures by the intermolecular polymerization of cyanides. Subsequently, the isoindoline structures were produced by linear growth of the triazine ring structures, followed by the final formation of phthalocyanine rings via the intramolecular polymerization [6,35,48]. Except for hydroxy groups, B-H bonds in QCB also provided active hydrogen atoms [47]. This could be explained by the following reasons: carbon and boron atoms in carborane both were six-ligand, obeying Hückel’s rule and exhibited aromaticity, which caused low energy of the B-H bond. The cage structure of carborane made the boron atoms on neighboring carbon atoms be slightly positive and prone to occur nucleophilic substitution reactions [28]. Therefore, when curing with QCB catalyzers, a large number of reaction intermediates were generated and the rate of cyclization of the intermediates was further enhanced. This also explained why there were two obvious exothermal peaks in the DSC spectra of BPh-Q_10_. Moreover, with further increased content of QCB, more B-H and hydroxyl groups produce more reaction intermediates, allowing triazine and phthalocyanine rings to be generated at lower temperatures, resulting in the advance of the second curing peak.

The mechanism for the curing of BPh monomers effectively catalyzed by QCB was further confirmed by pyrolysis GC-MS (Table 3). The volatile products of BPh-Q thermosets formed at 800 °C during curing were mainly composed of triazine ring and isoindoline structures. Moreover, the phthalocyanine ring structure was not detected in the pyrolysis products. In combination with the color of BPh-Q prepolymer and FTIR results, large amounts of triazine and isoindoline structures accompanied by trace amounts of phthalocyanine rings were produced during the curing reaction of BPh-Q. These results indicated that the polymerization of BPh was dramatically speeded up by introducing QCB with high catalytic activity and large number of active hydrogen atoms, and the triazine and isoindoline structures generated in the curing reaction allowed the polymerization of more cyanide groups.

### 3.4. Thermal and ThermoOoxidative Stability of BPh-Q and BPh-B Resins

**Figure 8:**
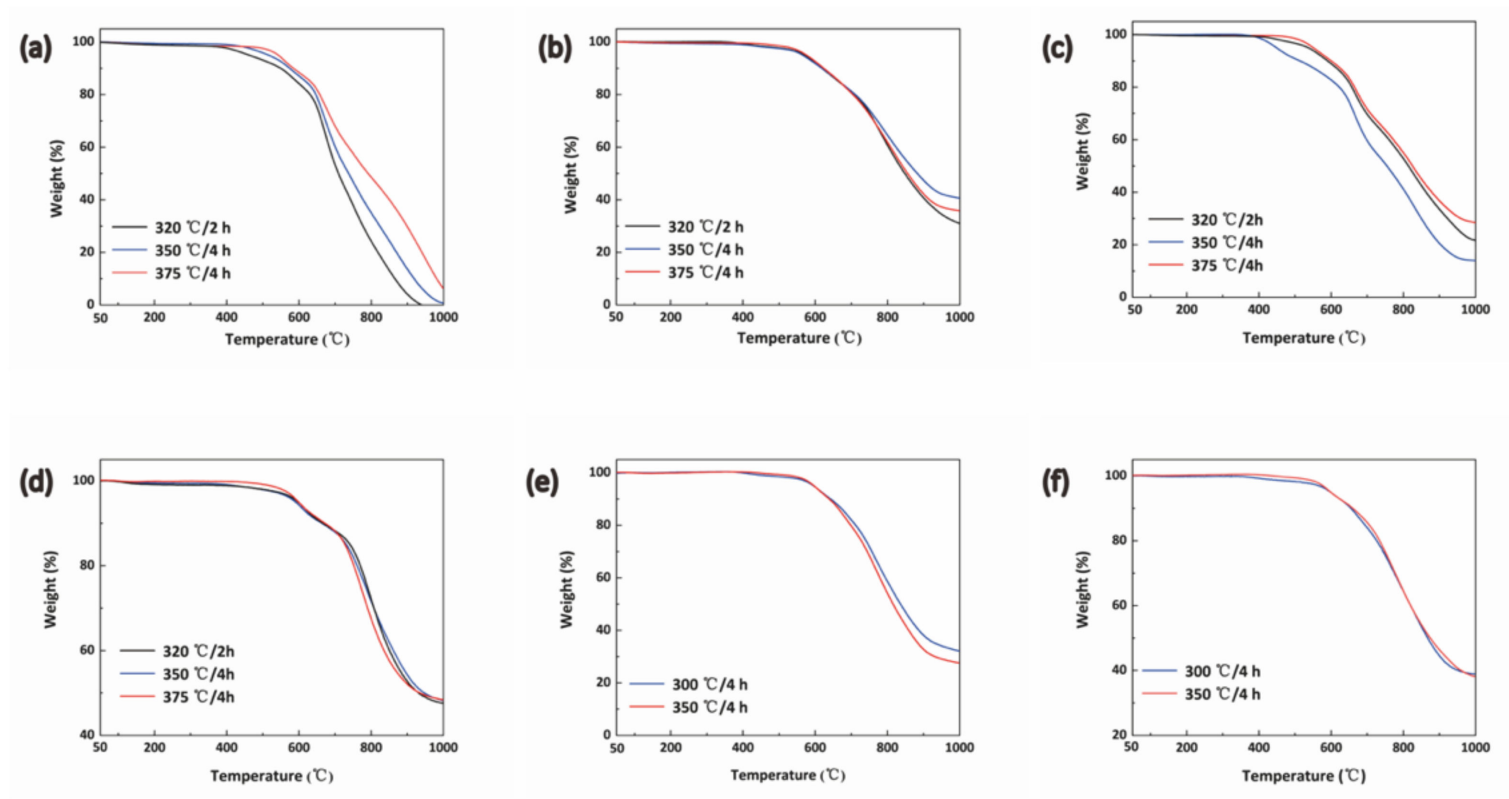
TGA curves of (**a**) BPh-B; (**b**) BPh-Q_5_; (**c**) BPh-Q_10_; (**d**) BPh-Q_15_; (**e**) BPh-Q_20_; (**f**) BPh-Q_30_ in air, the experimental temperature ranges from 50 to 1000 °C, with a heating rate of 10 °C/min (under nitrogen or air atmosphere) and a purge of 40 mL/min.

The thermal and thermo-oxidative stability of BPh-Q and BPh-B polymer was investigated by thermogravimetric analysis (TGA) at the temperature of 50–1000 °C under N_2_ and air atmosphere, respectively (Figure 8 and Figure S5 and Table 4). In N_2_ atmosphere, T_d5_ were 487 °C and 538 °C or BPh-B after curving at 320 °C and 375 °C, respectively, in which the difference was induced by crosslinking density. In addition, T_d5_ of BPh (prepolymerized with 1,3-bis(3-aminophenoxy)benzene) cured to 375 °C is only about 500 °C, and the char yield is about 60% [10]. While the T_d5_ (580 °C) increased by 43 °C and 80 °C for BPh-Q_10_ cured at 375 °C and the char yield remained 80%.

This improvement in the thermal stability upon adding QCB could be explained by the more effective catalytic activity of QCB than that of BPA, leading to complete curing of the cyano groups at a lower temperature. In the oxidative environment, BPh-B thermosets were completely burned out. By comparison with BPh-Q_5_, BPh-Q_10_ and BPh-Q_15_ showed better thermal and thermal oxidation stability at the same heat treatment temperature and treatment time (Figure 8). Meanwhile, BPh-Q_10_, BPh-Q_15_, BPh-Q_20_ and BPh-Q_30_ exhibited almost the same thermal and thermal oxidation properties at three curing temperatures. It is indicated that the cure endpoint of BPh-Q was 320 °C, while that of BPh-B was 375 °C. This result could be attributed to the higher reaction degree of BPh-Q_10_, BPh-Q_15_, BPh-Q_20_ and BPh-Q_30_ thermosets compared to BPh-B and BPh-Q_5_ thermosets induced by the accelerated curing reaction via increasing the content of B-H and -CH_3_OH in BPh-Q. Besides, T_d5_ of BPh-Q showed similar values in air and nitrogen atmosphere, suggesting its excellent thermo-oxidative stability. Particularly, the T_d5_ of BPh-Q_15_ reached as high as 597 °C, and the char yield at 1000 °C was 48.40% in air atmosphere, indicating that QCB itself could also greatly improve the thermal stability and thermal oxidation stability of BPh. However, the thermal and thermo-oxidative stability of thermosetting resin is also related to crosslinking density [49]. When the content of QCB is greater than 20 wt%, a large amount of B-H and hydroxyl in BPh-Q reacted with cyano groups, which reduced the crosslinking density of the cured product and led to a certain decrease in the temperature resistance of BPh-Q.

**Figure 9:**
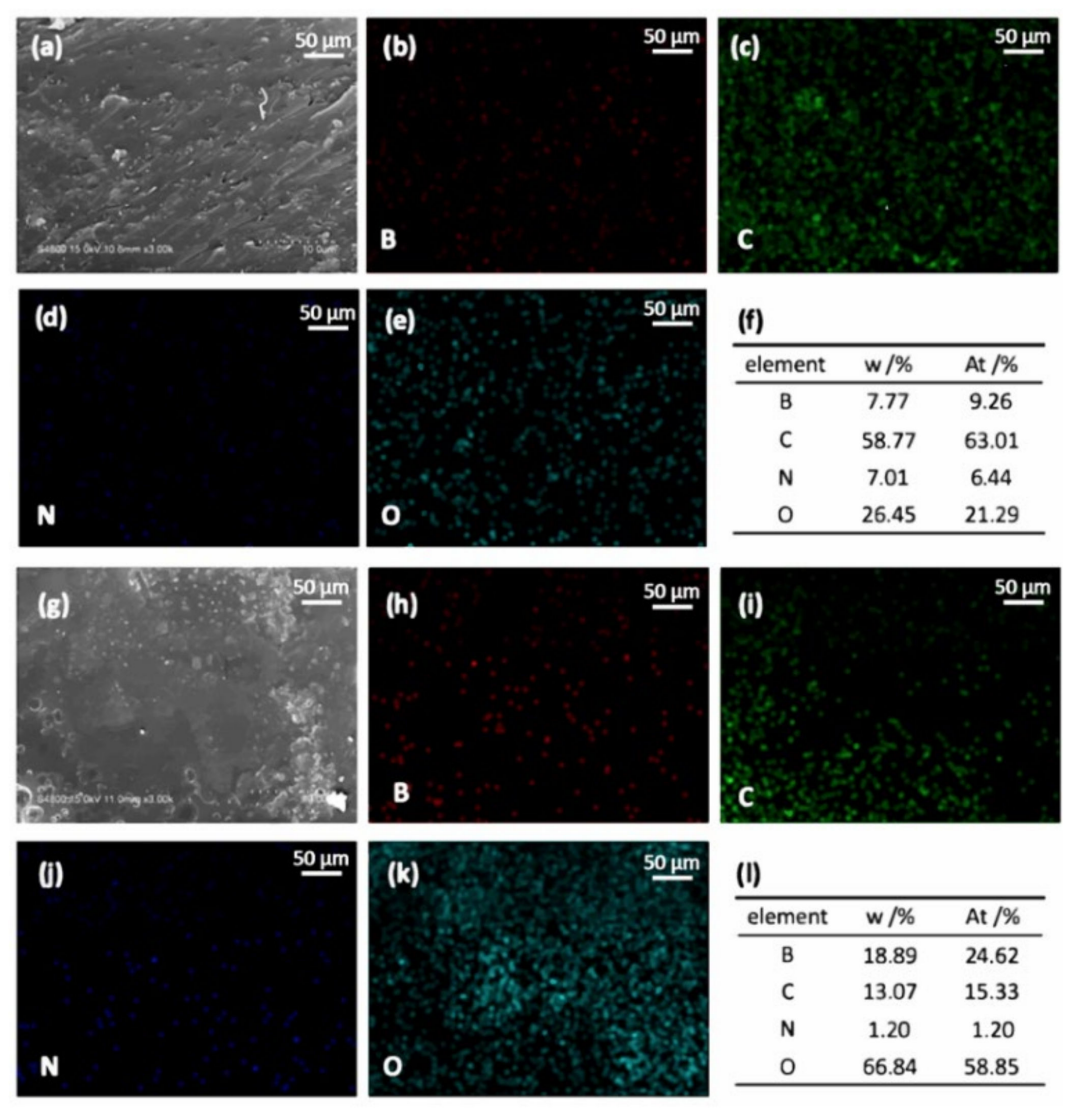
Micro morphology and energy spectrum analysis of BPh-Q_10_ thermosets before and after thermal oxidation treatment, (**b**–**e**) the corresponding EDS elemental mapping images before thermal oxidation treatment, (**a**,**g**) SEM of BPh-Q_10_ thermosets before and after thermal oxidation treatment, (**f**,**l**) the mass percentage of the surface element before and after thermal oxidation treatment and (**h**–**k**) the corresponding EDS elemental mapping images after thermal oxidation treatment. The gold spray time was 30 s.

The BPh-Q_10_ thermosets ablated before and after the TGA tests (in air) were further analyzed by EDS (Figure 9), and obvious differences were observed on the surface of the thermosets before and after the thermal oxidation treatment. Specifically, the contents of O and B elements were increased upon the oxidation treatment. Furthermore, the specific reactive C and N elements were gradually reacted with oxygen and transformed into C/O and N/O compounds. At the same time, B elements were reacted with oxygen to produce a B_2_O_3_ film on the surface of the thermoset, thereby hindering the erosion of the matrix by oxygen and heat flow, and prevents further decomposition of the internal resin [36]. Therefore, the addition of QCB improved the temperature resistance and thermal oxidation properties of the BPh thermosets due to the formation of boron oxide thin layer with excellent thermal stability on the surface of the thermosets.

**Figure 10:**
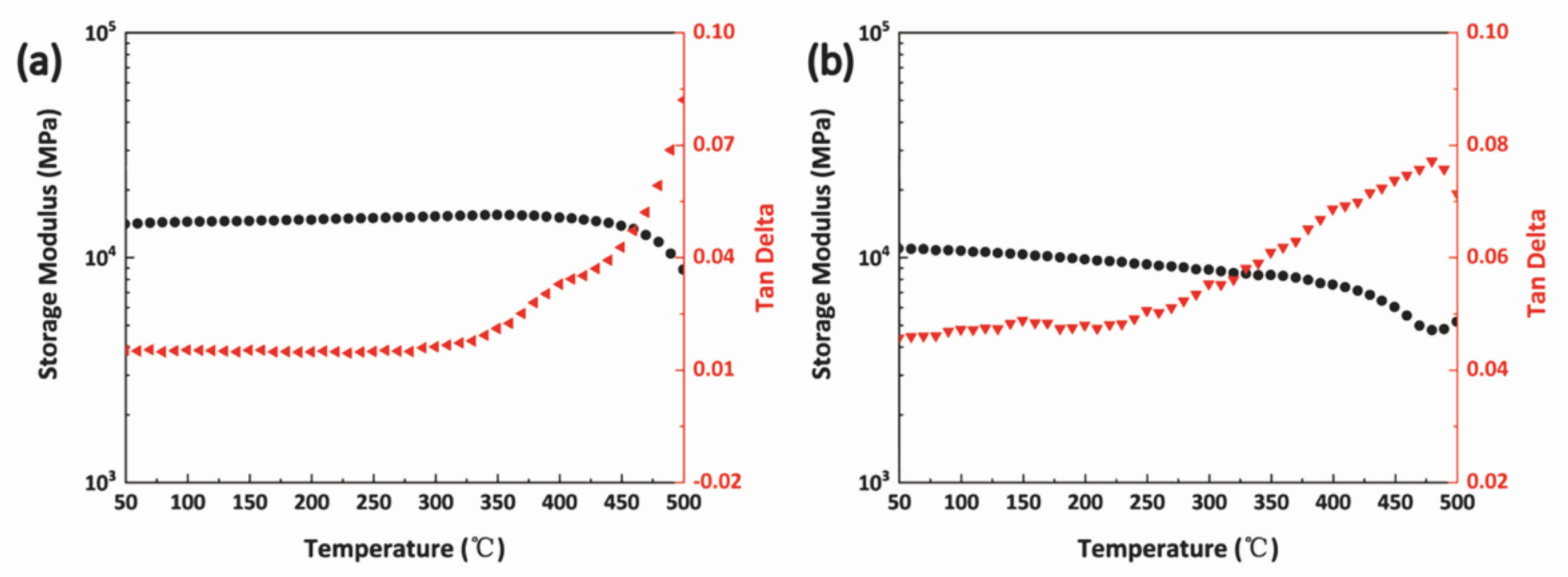
DMA curves of (**a**) BPh-Q_10_ and (**b**) BPh-B thermosets. The heating rate is 5 °C/min and the frequency is 1.0 Hz.

Dynamic mechanical analysis (DMA) is regarded as an effective test to characterize the thermo-mechanical properties of BPh-Q and BPh-B (Figure 10). With the increase of temperature, the storage modulus of the resins hardly changed, suggesting both the resins have qualified thermo-mechanical properties. Notably, the storage modulus of BPh-Q_10_ was as high as 15.6 GPa, while BPh-B was 10.9 GPa, indicating that the BPh-Q_10_ was more rigid. Moreover, the tan δ of BPh-Q_10_ did not form a complete peak in DMA measurement even when the temperature is near 500 °C, compared with BPh-B (Tg is 474 °C). It can be concluded from the comparison with BPh-B that the prepolymerization with QCB does increase the service temperature of BPh.

## 4. Conclusions

In summary, A distinctive BPh-Q resin was successfully prepared by the prepolymerization of BPh monomers and QCB. Their curing behavior, mechanism and processability were studied and discussed. DSC curve showed that there was a curing peak of BPh-Q at 260 °C, and the exothermic peak at 320 °C moved to lower temperature with the increase content of QCB. The results of experimental and theoretical models revealed that the B-H bond of QCB could effectively promote the curing reaction of phthalonitrile via Markovnikov addition reaction, thereby producing BPh-Q with good solubility, fast curing speed and low curing temperature. Furthermore, the thermosets of BPh-Q showed high thermal and thermo-oxidative resistance, and BPh-Q could be cured completely at 320 °C. The Tg of the cured resin is higher than 500 °C, and the storage modulus almost does not decrease, indicating that the resin has excellent thermo-mechanical properties. BPh-Q prepolymer is expected to be employed for further applications in, for example, high-temperature resistant composite parts that require low-temperature curing. Besides, this work may provide new insights into the modification of cyanide resin promoters and pave a way for the preparation of organic–inorganic hybrid resin.

## Figures and Tables

**Table 1:** Solubility of BPh prepolymers.

Samples	Solubility ^a^					
	NMP ^b^	DMF	DMAc	THF	Acetone	CHCl_3_
BPh-Q_5_	++	++	++	++	++	++
BPh-Q_10_	++	++	++	++	++	++
BPh-Q_15_	++	++	++	++	++	++
BPh-Q_20_	++	++	++	++	++	++
BPh-Q_30_	++	++	++	++	++	++
BPh-B	++	++	++	+-	+−	+
BPh	++	+	+	−	−	+

^a^ Solubility: ++: soluble in room temperature; +: soluble on heating; +−: partially soluble on heating; −: insoluble. ^b^ NMP: N-Methyl pyrrolidone; DMF: N,N-Dimethylformamide; DMAc: N,N-Dimethylacetamide; THF: Tetrahydrofuran, the solid content is 20%.

**Table 2:** Curing degree of BPh-Q prepolymers.

Samples	ΔH (J/g)		Curing Degree (%)
	ΔH_total_	ΔH_res_	
BPh-Q_5_	455.25	10.88	97.61
BPh-Q_10_	427.83	6.59	98.46
BPh-Q_15_	567.69	6.53	98.85
BPh-Q_20_	525.55	9.09	98.27
BPh-Q_30_	462.93	7.73	98.33

**Table 3:** Composition of BPh-Q_10_ cracking products at 800 °C.

No.	Crack Products at 800 °C Volatile Products	Content (%)
1	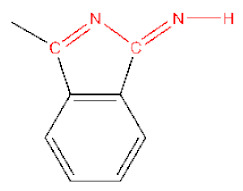	19.00
2	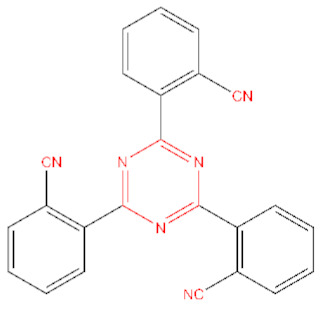	16.59
3	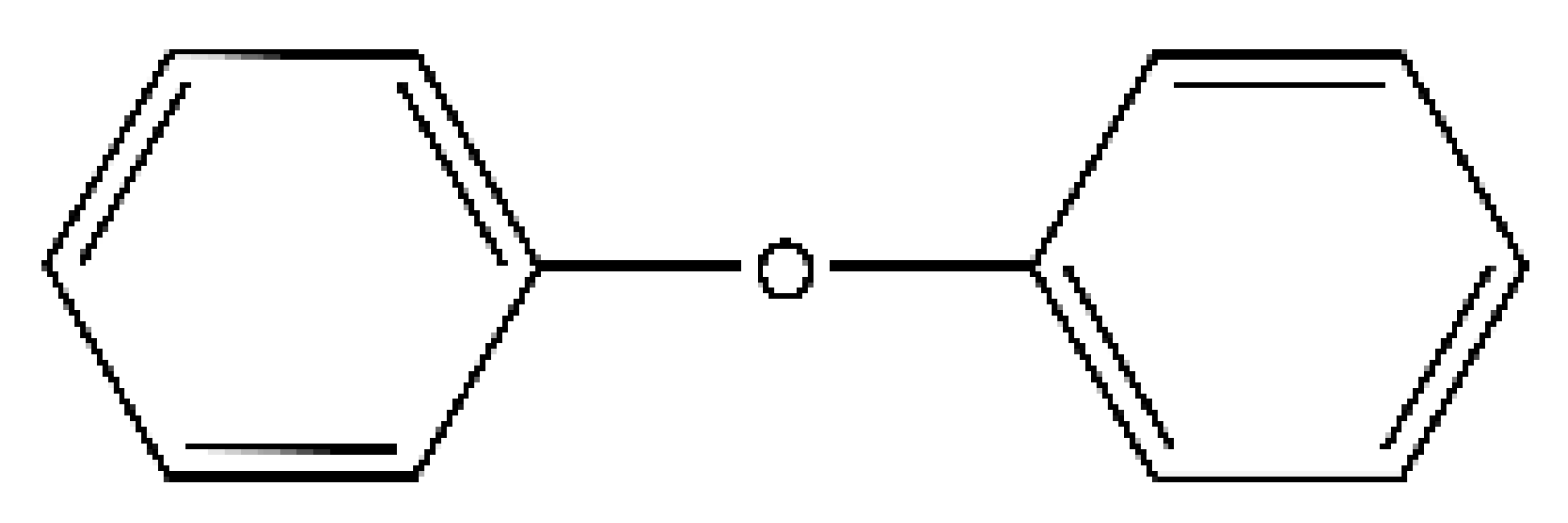	13.94
4	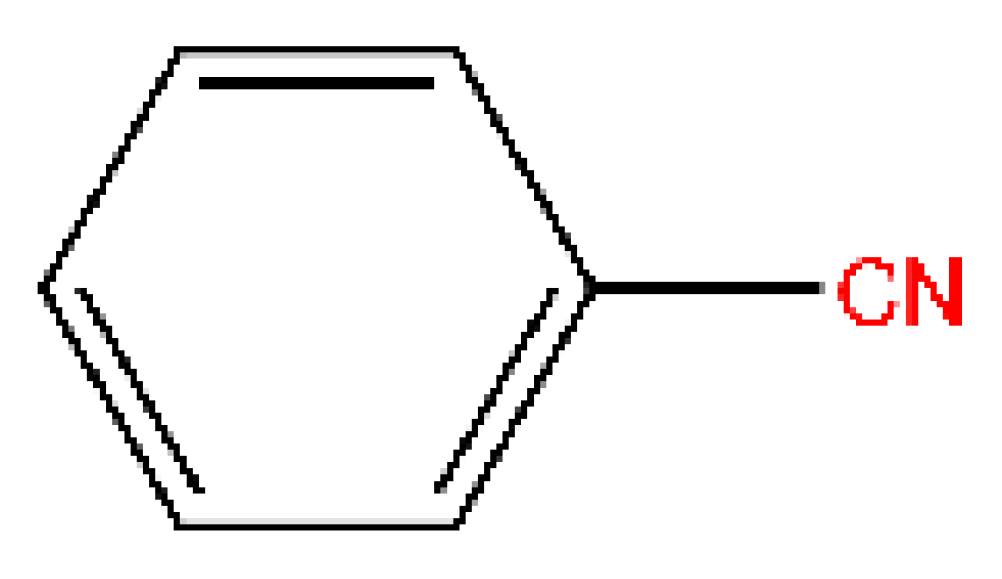	7.28
5	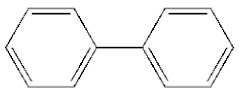	4.72
6	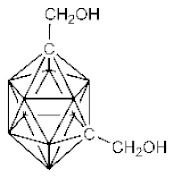	3.14

**Table 4:** Thermal stabilities of BPh-Q and BPh-B treated at different temperatures.

Sample	Curing Conditions	In N_2_		In Air	
		T_d5_ (°C)	Char Yield (%)	T_d5_ (°C)	Char Yield (%)
BPh-B	320 °C/2 h	487	67.41	462	0.00
	350 °C/4 h	492	72.60	516	0.80
	375 °C/4 h	538	75.80	542	6.40
BPh-Q_5_	320 °C/2 h	492	73.53	460	13.97
	350 °C/4 h	534	74.79	537	21.78
	375 °C/4 h	535	73.86	547	28.45
BPh-Q_10_	320 °C/2 h	570	79.30	568	31.10
	350 °C/4 h	580	80.30	565	40.60
	375 °C/4 h	578	79.50	575	35.90
BPh-Q_15_	320 °C/2 h	590	84.21	591	48.22
	350 °C/4 h	596	82.09	597	47.61
	375 °C/4 h	609	84.20	597	48.40
BPh-Q_20_	320 °C/4 h	594	83.31	595	32.20
	375 °C/4 h	592	83.23	596	28.64
BPh-Q_30_	320 °C/4 h	613	84.87	598	38.93
	375 °C/4 h	615	86.52	598	38.17

## Supplementary Materials

The following are available online at https://www.mdpi.com/article/10.3390/polym14010219/s1, Figure S1: DSC curves of QCB; Figure S2: DSC curves of BPh-Q prepolymers after curing; Figure S3: Rheological curve of BPh monomer; Figure S4: FTIR spectrum of BPh-B prepolymer; Figure S5: TGA curves of (a) BPh-B; (b) BPh-Q_5_; (c) BPh-Q_10_; (d) BPh-Q_15_; (e) BPh-Q_20_; (f) BPh-Q_30_ in N_2_, the experimental temperature ranges from 50 to 1000 °C, with a heating rate of 10 °C/min (under nitrogen or air atmosphere) and a purge of 40 mL/min; Table S1: Key bond lengths of transition states (TS_AM_ and TS_M_).
