# Supplementary material for: Catalytic Polymerization of Phthalonitrile Resins by Carborane with Enhanced Thermal Oxidation Resistance: Experimental and Molecular Simulation
Source: Polymers (Basel). 2022 Jan 5;14(1):219. doi: 10.3390/polym14010219 (PMC8747195; doi:10.3390/polym14010219)
Supplement: Supplementary file 1 [file polymers-14-00219-s001.zip › Table S1.pdf]

Table S1. Key bond lengths of transition states (TS_{AM} and TS_{M}).

Structure	Bond length (Å)	
TS_{AM}	C-N	1.17
	B-N	1.68
	C-H	1.18
	B-H	1.45
TS_{M}	C-N	1.28
	B-N	2.59
	C-H	1.90
	B-H	2.59
