# Supplementary figures and images for: Catalytic Polymerization of Phthalonitrile Resins by Carborane with Enhanced Thermal Oxidation Resistance: Experimental and Molecular Simulation
Source: Polymers (Basel). 2022 Jan 5;14(1):219. doi: 10.3390/polym14010219 (PMC8747195; doi:10.3390/polym14010219)

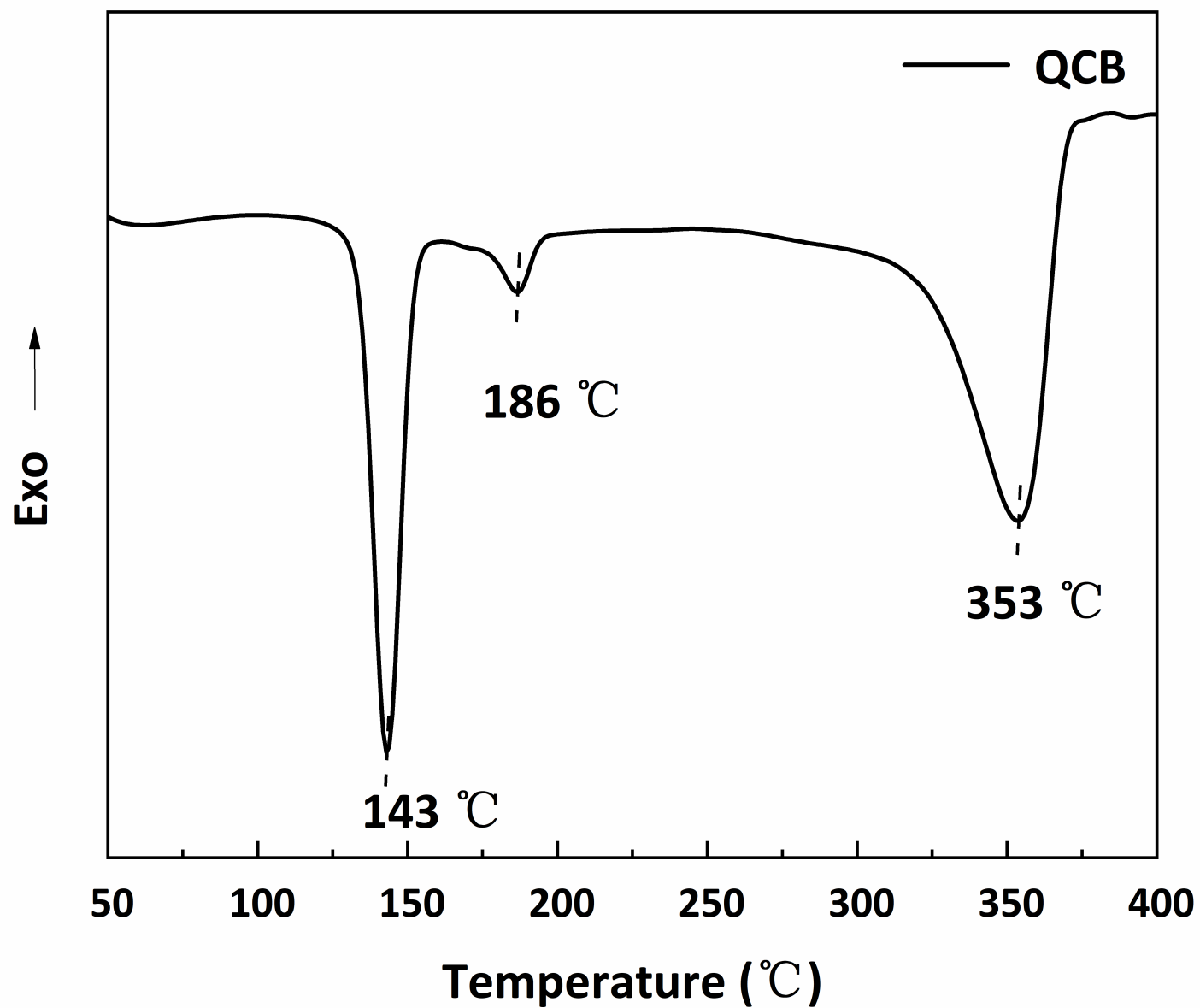

Supplement: Supplementary file 1 [file polymers-14-00219-s001.zip › Figure S1.pdf]

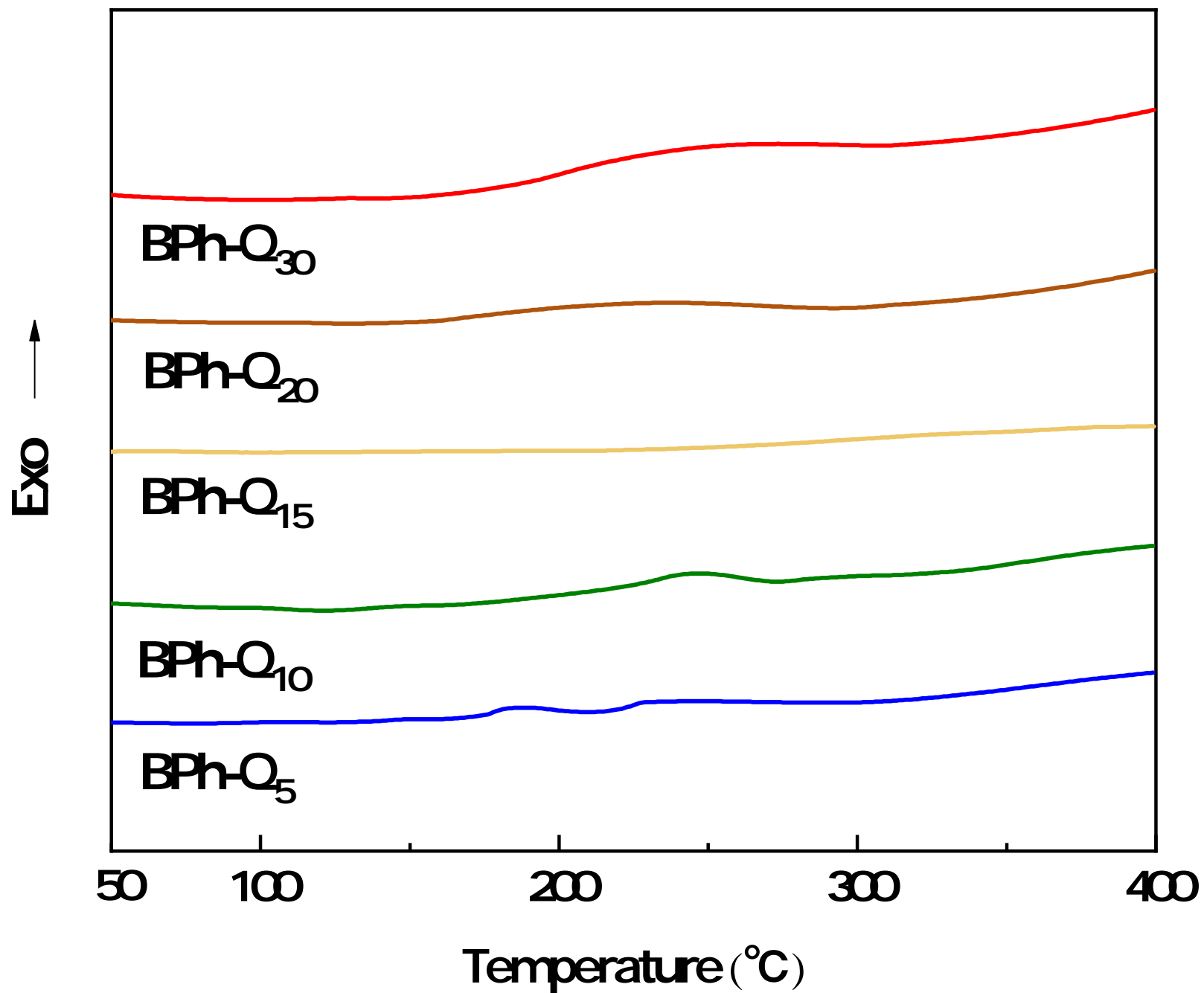

Supplement: Supplementary file 1 [file polymers-14-00219-s001.zip › Figure S2.pdf]

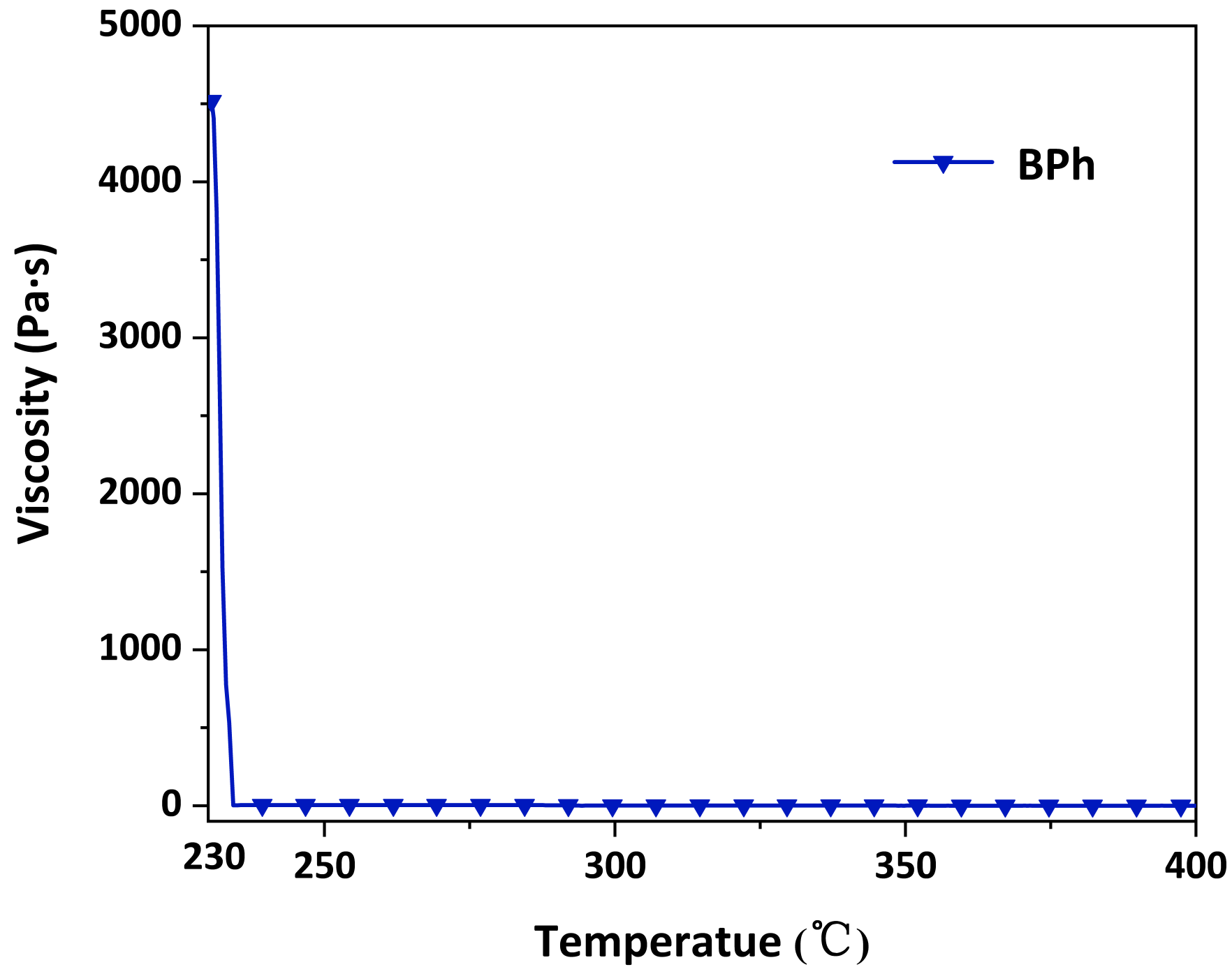

Supplement: Supplementary file 1 [file polymers-14-00219-s001.zip › Figure S3.pdf]

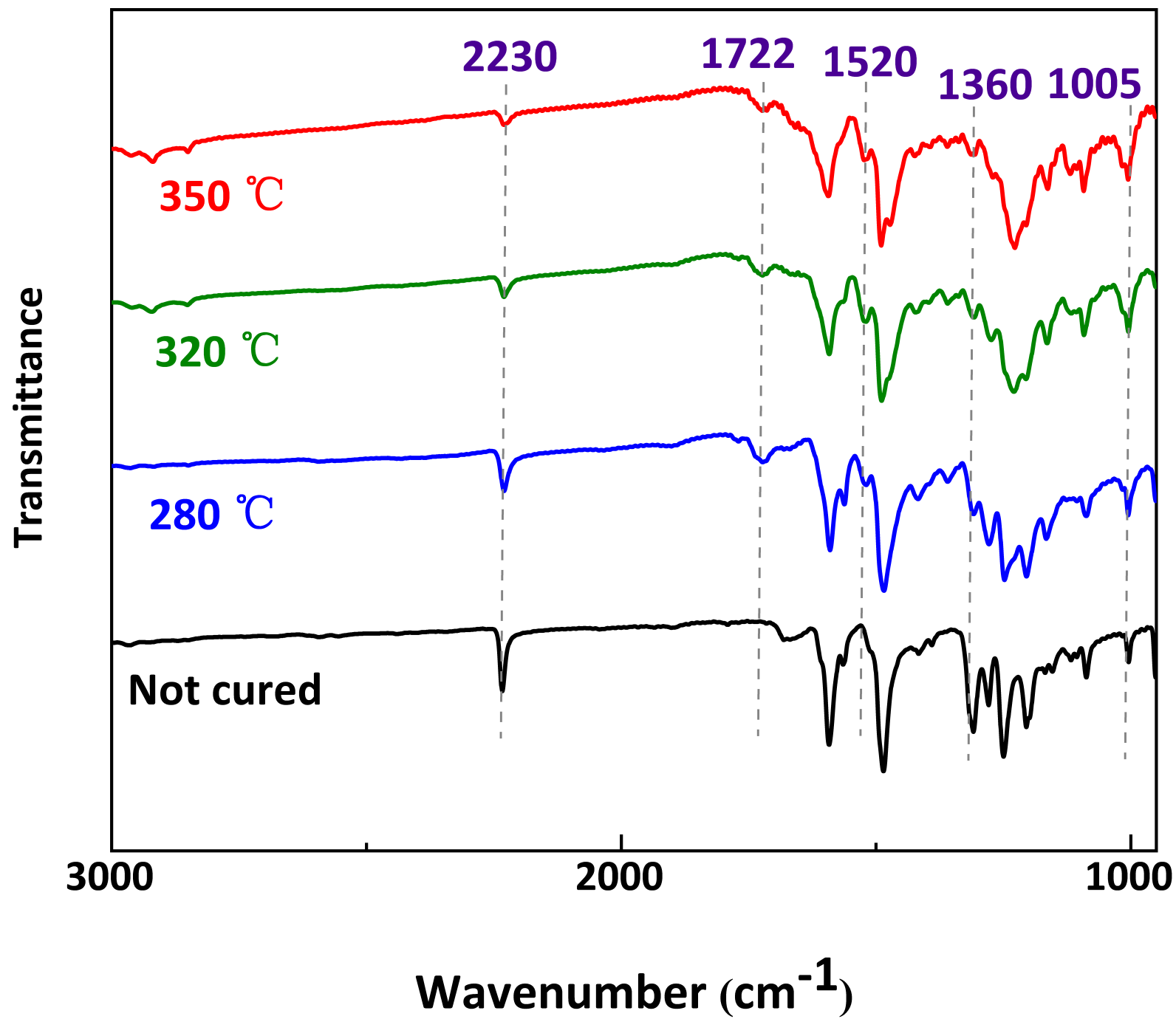

Supplement: Supplementary file 1 [file polymers-14-00219-s001.zip › Figure S4.pdf]

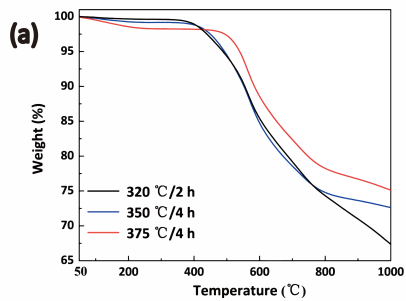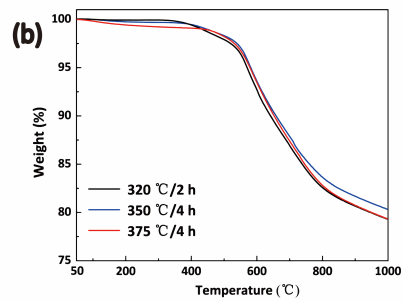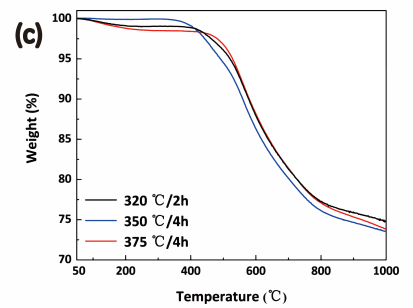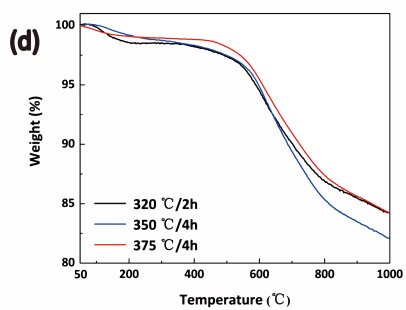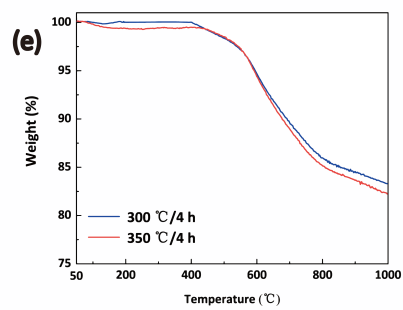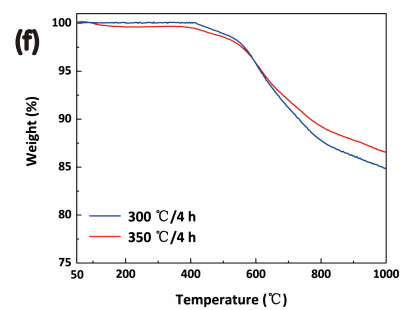

Supplement: Supplementary file 1 [file polymers-14-00219-s001.zip › Figure S5.pdf]
